# Theory of random measurement errors: concepts, uses, and misuses

**DOI:** 10.2478/aiht-2025-76-3977

**Published:** 2025-06-30

**Authors:** Branimir Zauner, Branko Petrinec, Tomislav Bituh, Saša Ceci, Nikola Volarić, Aleksandar Včev, Andrea Vukoja, Dinko Babić

**Affiliations:** Institute for Medical Research and Occupational Health, Zagreb, Croatia; Ruđer Bošković Institute, Zagreb, Croatia; Josip Juraj Strossmayer University of Osijek Faculty of Dental Medicine and Health, Osijek, Croatia; University Hospital Centre Osijek, Croatia

**Keywords:** mean value, relative uncertainty, statistics, uncertainty of the mean, nepouzdanost srednje vrijednosti, relativna nepouzdanost, srednja vrijednost, statistika

## Abstract

We present an overview of the theory of random measurement errors, focusing on the underlying concepts rather than on a strict mathematical formulation. Although the related literature is extensive, one can frequently encounter partly or completely wrong usages of the theory. In many cases, these misuses stem from incomplete understanding of the basic principles. Our presentation is based on a discussion on similarities and differences between this theory and statistics, as they are used differently in analysing the results of an experiment. In statistics, the central parameters are the mean and standard deviation, which are related to a given statistical distribution. In the theory of random measurement errors, the mean has a different meaning, representing the best estimate of the true value of a measured quantity. The second parameter of importance is not standard deviation but the uncertainty of the mean, which sets the probability that the true value lies in a given interval around the mean. These conceptual differences are seldom pointed out, which sometimes results in doubtful or wrong analyses and presentations of measurement results. We illustrate our theoretical considerations with examples of proper and improper use of the theory.

## Abstract (hr)

Predstavljamo pregled teorije slučajnih mjernih pogrešaka, fokusirajući se više na ishodišne koncepte nego na striktnu matematičku formulaciju. Iako je literatura o toj temi opsežna, često se susreću djelomično ili potpuno pogrešna korištenja spomenute teorije. U mnogo su slučajeva pogrešna korištenja povezana s nepotpunim razumijevanjem osnovnih principa. To nas motivira da svoju prezentaciju temeljimo na raspravi o sličnostima i razlikama između navedene teorije i statistike, koje se u analizi eksperimentalnih rezultata koriste drugačije. Statistički centralni parametri su srednja vrijednost i standardna devijacija, koje su vezane uz danu statističku raspodjelu. Srednja vrijednost u teoriji slučajnih mjernih pogrešaka ima drugačije značenje i najbolja je procjena prave vrijednosti mjerene veličine. Drugi važan parametar nije standardna devijacija nego nepouzdanost srednje vrijednosti, koja određuje vjerojatnost da prava vrijednost leži u danom intervalu oko srednje vrijednosti. Te konceptne razlike rijetko se naglašavaju, što ponekad vodi do dvojbenih ili pogrešnih analiza i prezentacija mjernih rezultata. Naša teorijska razmatranja dopunjena su primjerima ispravnih i neispravnih korištenja spomenute teorije.

Before the Renaissance, it was not fully recognised that the understanding of a phenomenon could not be obtained solely by a qualitative description of what had been observed. However, it eventually became clear that observations required quantifications, i.e., that the final outcome of an experiment should be expressed in numbers. This gave rise to a rapid development of experimental techniques and mathematics required to analyse measurement results. In that, one of the central issues was how to quantitatively account for the uncertainties of numerical values that were assigned to given observables, and answering this question eventually resulted in the theory of random measurement errors (TRME).

TRME was developed in the 19^th^ century by mathematicians and those experimental scientists who knew mathematics sufficiently well. In spite of the TRME being quite old, its spread though different fields of science and technology has been somewhat paradoxical, as the use of formulae has not always been accompanied by an understanding of their origin and meaning. For instance, at many universities, only some students are taught TRME in enough depth, whereas the others get acquainted with it briefly and superficially. It is also common that mathematics courses for students in many fields (e.g., social, medical, biological, or biotechnical sciences) are inadequate for coping with the specific mathematical language of TRME. In consequence, some researchers – usually from disciplines that are not mathematically oriented – cannot fully distinguish between statistics and TRME, which may diminish the quality of a presentation of research results.

After so many years of development, literature abounds with articles (1, 2), textbooks (3,4,5), guides (6), and other documents on TRME that nowadays appear on the Internet on a daily basis. The goal of this review is not to summarise or expand the information presented in these sources. Instead, we focus on clarifying the concepts from which TRME stems to clearly distinguish between descriptive statistics and the analysis of a measurement. Such presentations are not common in the literature, which often leads to inconsistent analyses of experimental results due to the lack of a clear picture of what TRME is.

## BASIC CONCEPTS OF STATISTICS

### Statistical distributions, frequency distributions, and their descriptive parameters

Statistics is a mathematical discipline dedicated to properties of systems in which a variable can assume different values. For instance, the height of a person can be a variable, and the corresponding statistical system is formed from measured heights within a group of people. Generally, there are two types of statistics. *A priori* statistics is dedicated to theoretical modelling which provides probabilities that a variable will assume given values. Mathematical formulae that describe these probabilities are called statistical distributions. They are sometimes named after those who derived them (e.g., Gaussian distribution, Poisson distribution, Bernoulli distribution) and sometimes contain mathematical terminology (e.g., gamma distribution, exponential distribution, log-normal distribution). *A posteriori* statistics focuses on measured occurrences of values, which results in frequency distributions. While frequency distributions always contain discrete numbers, statistical distributions can be expressed either through pairs of discrete numbers (discrete distributions) or as functions (continuous distributions). Comparisons of frequency distributions and statistical distributions are common and may lead to a deeper understanding of given experimental results.

Let us consider random variables, i.e., those which assume different values solely due to the corresponding probabilities of their appearances. Suppose that a variable *x* can assume values *x*_1_,..., *x*_n_ and that the probability of *x* = *x*_*i*_ is equal to *p*(*x*_*i*_), where 0 ≤ *p*(*x*_*i*_) ≤ 1. The set of pairs [*x*_*i*_, *p*(*x*_*i*_)] then forms a discrete statistical distribution of the variable *x*, satisfying the condition $\sum_{i=1}^{n}p(x_{i})=1$ (which means that the probability that *x* will assume any value is 100 %). As explained above, the values of *p*(*x*_*i*_) are calculated theoretically (*a priori* statistics). We can also carry out *N* measurements of *x* and record, for each *i*, the number *f*(*x*_*i*_) (called absolute frequency) of the appearance of *x*_*i*_. Using this, we can define *r*(*x*_*i*_) = *f*(*x*_*i*_)/*N* (called relative frequency), which represents the experimental probability (*a posteriori* statistics) of the appearance of *x*_*i*_, satisfying $\sum_{i=1}^{n}f(x_{i})=N$ and, consequently, $\sum_{i=1}^{n}r(x_{i})=1$ . The set of pairs [*x*_*i*_,*r*(*x*_*i*_)] forms a frequency distribution.

Statistical properties of a system are usually expressed through descriptive statistical parameters, derived from statistical moments *m_l_* and *M_l_* (the *l*th raw moment and central moment, respectively), which are calculated as

[1] $$m_{l}=\sum_{i=1}^{n}x_{i}^{l}p\left(x_{i}\right)$$

and,

[2] $$M_{l}=\sum_{i=1}^{n}{\left(x_{i}-m_{1}\right)}^{l}p\left(x_{i}\right)$$

For a frequency distribution, the *p*(*x*_*i*_) in the above equations (and henceforth), should be replaced with *r*(*x*_*i*_).

In most cases, four parameters are used to describe a distribution. The first is *x̄* = *m*_1_, called the expectation or the mean (value) of a statistical distribution or a frequency distribution, respectively. The second is variance *V* = *σ*^2^ = *M*_2_, which is basically the mean squared deviation of *x* from *x̄*. The third is skewness *α*_3_ = *M*_3_/*σ*^3^, which accounts for the asymmetry of a distribution. The fourth is kurtosis *α*_4_ = *M*_4_/*σ*^4^, which is a measure of the tailedness of a distribution.

The probability that *x* ≥ *x*_*a*_ and *x* ≤ *x*_*b*_ is calculated as follows:

[3] $$P\left(x_{a}\leq x\leq x_{b}\right)=\sum_{i=a}^{b}p\left(x_{i}\right)$$

As mentioned before, there are also continuous statistical distributions besides the discrete ones. In this case, *x* is a continuous variable, and this changes the required mathematics. We define a continuous function *g*(*x*) ≥ 0, called probability distribution, with a property that the probability of *x* being in the interval (*x*, *x* + *dx*) is given by *g*(*x*)*dx*. This means that ∫ *g*(*x*)*dx* = 1 over the range where *x* is defined and that [3] changes into

[4] $$P(x_{a}<x<x_{b})=\int_{x_{a}}^{x_{b}}g\left(x\right)\mathrm{dx}$$

Graphically, this is the area under *g*(*x*) between *x* = *x*_*a*_ and *x* = *x*_*b*_. Equations [1] and [2] change as well, by replacing *x*_*i*_ with *x* and $\sum_{i=1}^{n}p(x_{i})$ with ∫ *g*(*x*)*dx* = 1, where the integration is carried out over the range where x is defined. The meanings of *x̄*, *σ*, *a*_3_ and *a*_4_ remain the same.

There are two additional parameters which are frequently used to describe the main properties of a distribution. The first is the median η of a distribution, which is defined by *P*(*x* < η) = *P*(*x* > η) = 1/2. The second is the most probable value *ν* of *x*, defined as the point at which there is a maximum of *p*(*x*_*i*_) or *P*(*x*).

### Gaussian distribution as the basis of TRME

Gaussian or normal distribution is arguably the best-known statistical distribution, being applicable in numerous fields of science and technology. Since TRME relies on it, we shall briefly discuss its main properties. This is a continuous statistical distribution defined as:

[5] $$g(x)=\frac{1}{\sigma \sqrt{2\pi }}e^{-\frac{{\left(x-\bar{x}\right)}^{2}}{2\sigma^{2}}};-\infty <x<\infty$$

**Figure 1:**
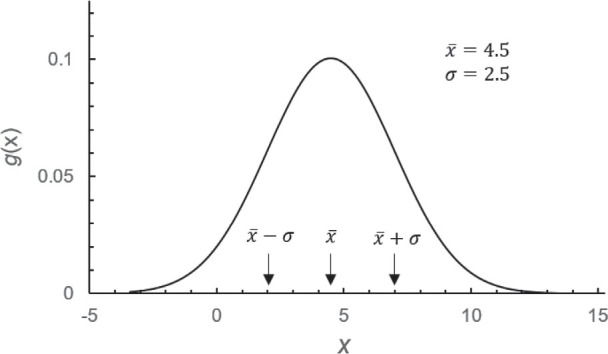
Probability distribution *g*(*x*) of a random variable *x* for the Gaussian distribution with *x̄* = 4.5 and *σ* = 2.5

As shown in Figure 1, *g*(*x*) is a symmetrical, bell shaped curve peaked at *x̄*. At = *x̄* ± *σ*, there are inflexion points, i.e., the curvature of *g*(*x*) changes from convex to concave. The shape of the curve is completely determined by *x̄* and *σ*, whereas *α*_3_ = 0, *α*_4_ = 3, and η = *ν* = *x̄*.

One of the properties of Gaussian distribution is that *P*(*x* – *kσ* < *x* < *x̄* + *kσ*) is about 0.68, 0.95, and 0.98 for *k* = 1, 2, and 3, respectively. Of course, one can choose any *k* (called coverage factor) and calculate the corresponding *P*, but the above integer values are most commonly used in TRME.

## STATISTICAL ANALYSES OF MEASUREMENT DATA

### Types of measurement errors

Measurement errors can be systematic or random. Systematic errors are specific of every measurement procedure and are usually manifested as offsets in measured results, e.g., when an instrument is showing some value in the absence of any input. These errors are not uncommon – and are solved by removing a given offset technically or analytically – but are not in the domain of TRME and are therefore not addressed in this review. TRME deals with random errors, which originate from limited accuracy of measurement instruments and procedures. Random errors do not produce offsets and are manifested as symmetric departures of measurement results from *x̄*, with *r*(*x*_*i*_) following closely the Gaussian distribution.

### TRME versus statistics

In its approach and mathematical methods, TRME is based on statistics. Hence, formulae used in TRME and statistics are similar and sometimes even the same, but their meanings may be quite different. Suppose that a quantity x is measured repeatedly n times. In TRME, *p*(*x*_*i*_) = 1/*n*, and Equation [1] gives

[6] $$\bar{x}=\frac{\sum_{i=1}^{n}x_{i}}{n}$$

while the use of Equation [2] results in

[7] $$\sigma^{2}=\frac{\sum_{i=1}^{n}{\left(x_{i}-\bar{x}\right)}^{2}}{n}$$

Let us first discuss *x̄*. We have used a general formula from statistics to calculate it and have used the same name (the mean). However, the meaning of *x̄* in TRME is not the same as in statistics, where *x̄* is simply the average value of *x*. In TRME, *x̄* is the best estimate for the true value *ξ* of *x*. Since *ξ* cannot be determined – due to the limitations of measurement instruments and procedures – taking *ξ* ≈ *x̄* is the best we can do. The central quantity in TRME is the uncertainty *u* in this approximation, so we can express a measurement result as

[8] $$\xi =\bar{x}\pm u$$

While different names are used for *u* (e.g., measurement uncertainty), its full name – uncertainty of the mean – reveals its meaning more directly. More clearly, we approximate *ξ* ≈ *x̄*, but Equation [8] actually says that *ξ* can be anywhere, with a given probability, in a symmetric interval around *x̄*. The distribution that determines this probability is Gaussian distribution peaked at *x̄*. This defines *x̄* as the most probable value of *ξ* and implies that we can use a coverage factor to set the related confidence level. As already said, it is common to choose *k* = 1, 2, or 3, but this is just for the sake of simplicity (as any value of *k* can be used).

Now we turn to the meaning and applicability of *σ* as given by Equation [7]. *σ* is called the standard deviation of the population. Again, it is useful to spell out the meaning of *σ*: the square root of the mean squared deviation of *x* from *x̄* for the complete population of a studied quantity. However, seldom can one collect data for an entire population, and n measurements then refer to a sample taken from all possible values of *x*. It can be shown, but this is outside the scope of this review, that the denominator in Equation [7] is actually the number of independent variables (degrees of freedom), which is reduced by unity in case of a sample that is smaller than the entire population. Hence, the variance of a sample is given by

[9] $$s^{2}=\frac{\sum_{i=1}^{n}{\left(x_{i}-\bar{x}\right)}^{2}}{n-1}=\frac{n}{n-1}\sigma^{2}$$

Since *σ* and *s* represent the square roots of the mean squared deviations of *x* from *x̄* for an entire population and a sample, respectively, which of them should be used as *u* that enters Equation [8]? Shall we prefer the use of *s*, because we normally measure restricted datasets, that is, samples? The answer to this question is: neither *σ* nor *s* can be used as *u* in Equation [8]. Just read their names: *σ* and *s* are measures of the mean squared deviation of *x* from *x̄* in a set of measurements, nothing more, and *u* is fundamentally different – it is the uncertainty of the approximation *ξ* ≈ *x̄*. This is why it is important to keep in mind the full names of *σ*, *s*, and *u*, because the common short names do not reveal clearly the meaning of these quantities. Of course, short names are welcome, but one should not forget what is behind them. For the rest of this review, we shall focus on *u* and discuss it from the perspective of its proper meaning, but shall simply call it “uncertainty”.

### Propagation of uncertainty

Before we address *u* more closely, we ought to discuss one of its important properties. Let us suppose that we measure quantities *q*_1_, *q*_2_,…, *q*_*z*_ directly and obtain *ξ*_*j*_ = *q̄_j_* ± u_*j*_ for all 1 ≤ *j* ≤*z*. Suppose also that there is a quantity *y* = *y*(*q*_1_, *q*_2_,…, *q*_*z*_) that has not been measured directly but can be calculated from *q*_1_, *q*_2_,…, *q*_*z*_ by using a formula. Having *ȳ_j_* and *u_j_*, can we find *ȳ* and *u_y_*, the mean and uncertainty of *y*, respectively? Yes, we can, using the following equations:

[10] $$\bar{y}=y\left({\bar{q}}_{1},{\bar{q}}_{2},\;\ldots ,{\bar{q}}_{z}\right)$$

and

[11] $$u_{y}^{2}=\sum_{j=1}^{z}{\left(\frac{\partial y}{\partial q_{j}}\right)}_{q_{j}={\bar{q}}_{j}}^{2}u_{j}^{2}$$

Equation [10] simply says that *ȳ* is obtained by inserting the values of *q̄_j_* (for all *j*) in the formula that relates *y* and *q̄*_*j*_. Regarding Equation [11], although it can be addressed rigorously from a mathematical point of view, we choose to discuss it more intuitively. One can see that $u_{y}^{2}$ is a sum of squared uncertainties $\left(u_{j}^{2}\right)$ of individual contributors to *y* and that each $u_{j}^{2}$ is multiplied by a weight factor ${\left(\partial y/\partial q_{j}\right)}_{q_{j}={\bar{q}}_{j}}^{2}$ that determines how much a change of *q*_*j*_ affects *y*. Partial derivatives must be squared in order to conserve dimensional consistency. If we take a look at the expression for *σ*^2^ = *M*_2_ (see Equation [2]), we can see that *σ*^2^ is also calculated as a weighted sum of squared individual deviations from *x̄*, the weight factors being *p*(*x*_*i*_). The mathematical similarity between the equations for *σ*_2_ and $u_{y}^{2}$ is not surprising, because both of these quantities are cumulative deviations that result from weighted individual contributions.

### Uncertainty of the mean

Equation [6] shows that *x̄* is a function of *x*_1_,…, *x*_n_, where each *x*_*i*_ has an uncertainty *u_i_*. Therefore, we can calculate uncertainty of the mean, i.e., *u* = *u*_*x̄*_, by using Equations [6] and [11] together with the symmetries *y* ↔ *x̄* and *q_i_* ↔ *x*_*i*_. This results in

[12] $$u^{2}=u\frac{2}{x}=\sum_{i=1}^{n}{\left(\frac{\partial \bar{x}}{\partial x_{i}}\right)}^{2}u_{i}^{2}=\sum_{i=1}^{n}\frac{1}{n^{2}}u_{i}^{2}$$

Since *x*_*i*_ is the result of a single measurement, the question arises as to what should be used for its (unknown) uncertainty *u_i_*. TRME assumes that *u_i_* is approximately the same for every *i* and equal to some *u_0_*, so that Equation [12] gives $u^{2}=u_{i}^{2}/n$ . The next assumption is that *u_0_* = *s*, so the use of Equation [9] results in

[13] $$u^{2}=\frac{\Sigma_{i=1}^{n}{\left(x_{i}-\bar{x}\right)}^{2}}{n\left(n-1\right)}=\frac{s^{2}}{n}=\frac{\sigma^{2}}{n-1}$$

We can see that *u*, *s*, and *σ* are interrelated, which is not surprising, given the statistical nature of TRME. However, while *s* and *σ* are indicators of how much individual measurement results depart from *x̄*, it is *u* which tells us how much *ξ* ≈ *x̄* is reliable. Moreover, the approximation *u_i_* = *u_0_* = *s* extends the meaning of *s* beyond simple statistics, since *s* in TRME is the average uncertainty of an individual measurement. With this interpretation, the relation *u*^2^ = *s*^2^/*n* becomes more understandable: if one carries out *n* measurements, the squared uncertainty will be reduced by a factor of 1/*n* in comparison with a single measurement.

### Weighted mean and its uncertainty

Equation [13] covers those experimental results where a direct measurement of an observable is repeated *n* times in order to reduce *u*. Equation [11] accounts for the *u* of a quantity which has not been measured directly but is calculated from several directly measured observables. The third frequent case is measurement results that have different statistical weights.

Suppose that a quantity *x* is measured using different methods or in different laboratories and that each of these measurements results in *ξ*_*j*_ = *x̄*_*j*_ ± *u_j_*. If one wants to compile all of these results and calculate their mean value and its uncertainty, how is this done properly? Since some of the results are more precise than the others, one should approach this problem by assigning a statistical weight *w_j_* to each *ξ*_*j*_.

To calculate the weighted mean, i.e., the mean 〈*x*〉 of *L* independent contributions, is straightforward, namely

[14] $$〈x〉=\frac{\Sigma_{j=1}^{L}w_{j}{\bar{x}}_{j}}{\Sigma_{j=1}^{L}w_{j}}$$

It is easy to understand the above expression by recalling that this is how one calculates grade point average if *x̄*j is a grade point and *w_j_* the number of its appearance.

We can now combine Equations [11] and [14] to derive the expression for the uncertainty 〈*u*〉 of 〈*x*〉, that is,

[15] $${〈u〉}^{2}=\sum_{j=1}^{L}{\left(\frac{\partial 〈x〉}{\partial {\bar{x}}_{j}}\right)}^{2}u_{j}^{2}=\frac{\Sigma_{j=1}^{L}w_{j}^{2}u_{j}^{2}}{{\left(\Sigma_{j=1}^{L}w_{j}\right)}^{2}}$$

In principle, *w_j_* can be defined in many different ways. On the other hand, $w_{j}=1/u_{j}^{2}$ is most commonly used, because this choice is applicable to many situations and assigns larger weights to results which are less uncertain. This definition of *w_j_* transforms Equation [14] into

[16] $$〈x〉=\frac{\Sigma_{j=1}^{L}\frac{{\bar{x}}_{j}}{u_{j}^{2}}}{\Sigma_{j=1}^{L}\frac{1}{u_{j}^{2}}}$$

and Equation [15] into

[17] $${〈u〉}^{2}=\frac{1}{\Sigma_{j=1}^{L}\frac{1}{u_{j}^{2}}}$$

### Maximum absolute uncertainty

There is another parameter which is sometimes calculated in order to obtain a more complete insight into measurement results. This parameter is called maximum absolute uncertainty and represents the maximum absolute departure of measurement results from *x̄*.

Its definition is

[18] $$\Delta x=|x_{i}-\bar{x}{|}_{\;\max\;}$$

Since Δ*x* is linear in the departure of *x*_*i*_ from *x̄*, the maximum absolute error Δ*y* of *y* = *y* = *y*(*q*_1_, *q*_2_,…, q_*z*_) contains linear terms, and Equation [11] changes into

[19] $$\Delta y=\sum_{j=1}^{z}{\left|\frac{\partial y}{\partial q_{j}}\right|}_{q_{j}={\bar{q}}_{j}}\Delta q_{j}$$

where the absolute values of *∂y/∂q_j_* ensure that every contribution to the sum is positive.

Equations [18] and [19] are also useful when repeated measurements always give the same result, for instance, when the length of an object is measured with a ruler of a limited precision. Does the fact that we always obtain the same number mean that there is no uncertainty? Obviously not. In this case, we should estimate the uncertainty in the sense of Δ*x*.

### Presentation of results

An analysis of experimental results provides numerical values of *x̄* (or *ȳ* or 〈*x*〉) and *u* (or Δ*x* or Δ*y* or 〈*u*〉). Usually, these values are used to calculate relative uncertainty

[20] $$R=\left(\frac{u}{\bar{x}}\;\text{or}\;\frac{u_{y}}{\bar{y}}\;\text{or}\;\frac{\Delta x}{\bar{x}}\;\text{or}\;\frac{\Delta y}{\bar{y}}\;\text{or}\;\frac{〈u〉}{〈x〉}\right)\times 100\%$$

which expresses uncertainty as a percentage of the mean. *R* is often included in the presentation of a result. For instance, if we obtain *x̄* = 4.7823 and *u* = 0.876, this is usually presented as

[21] ξ=4.8±0.9

The number of digits of *x̄* and *u* is smaller than the ones we have started from. The reason for that becomes clear if we recall the meaning of *u*: it sets a Gaussian interval around *x̄* where *ξ* lies with a probability given by the coverage factor. Hence, *u* is always rounded to one digit, as the second digit would represent the uncertainty of an uncertainty, and this would not make much sense. The only exception is when the second digit can be rounded to 5, and in this case, *u* is expressed with two digits (e.g., *u* = 0.014827 → *u* = 0.015). Once *u* is rounded properly, the number of digits of *x̄* (which are called significant digits) is set accordingly, to match the value of *u*. For example, *ξ* = 4.78±0.9 is not correct, because 0.08 (the third digit of the mean) is more than ten times smaller than the uncertainty 0.9. Similarly, *ξ* = 5±0.9 is also wrong, since *u* should not be more precise than *x̄*. Using too many digits for *u* and setting the number of significant digits of *x̄* wrongly are the most frequent mistakes in the presentation of a measurement result. There are no strict rules for the number of digits of *R*, because *R* is just an indicator and not a fundamental quantity. The number of digits should, however, be kept low in this case as well (e.g., 0.4 %, 7 %, 14 %, 112 %).

There are cases when the result contains very large or very small numbers, which can be solved by using the powers of ten and/or unit prefixes. For example, it is more elegant to write *ξ* = (4.8±0.9) μm or *ξ* = (4.8±0.9) × 10^−6^ m than *ξ* = (0.0000048±0.0000009) m.

## EXAMPLES OF USES AND MISUSES OF TRME

Let us continue by elaborating the above concepts with examples of uses and misuses of TRME. The uses refer to recognising the nature of given experimental results, applying proper calculations, and expressing the final result correctly. The misuses can be numerous, ranging from choosing a wrong formula to misinterpreting final results. We cannot address every possible (mis)use of TRME, but the below examples might be useful in handling numerous similar situations.

### Repeated direct measurements of a quantity

Suppose that a quantity is measured many times by the same instrument and the same method, with a goal to reduce *u*. For instance, one weighs an object repeatedly, every time obtaining a slightly different result for the mass. Provided that there are no systematic errors, these differences are the result of the imperfections of the scale and are distributed evenly around the mean value of the mass. In order to calculate the mean and its uncertainty, one uses Equation [6] and Equation [13], respectively. Then one selects the coverage factor to define *u* in the spirit of Equation [8], properly rounds *u* and then the mean, calculates *R*, and presents the result as demonstrated in Equation [21]. This is simplest use of TRME.

Even though *σ* and s can be calculated from the data, their roles are marginal. In fact, to use *σ* or *s* instead of *u* in Equation [8] is the most common mistake, i.e., a typical misuse of TRME.

### Indirect measurements

Suppose that we have to determine the density *ϱ* of a cylinder by measuring its mass *μ*, diameter *d*, and height *h*. Our measurement instruments are a calliper (with a precision of 0.05 mm) and a scale (with a precision of 1 mg). We measure *μ*, *d*, and *h* about ten times, observe small variations due to the imperfections of the instruments, and use Equations [6] and [13] to obtain *μ* = 28.903±0.004 g, *d* = 23.04±0.07 mm, and *h* = 12.42±0.04 mm.

We first calculate $\bar{\rho }=4\bar{\mu }/{\bar{d}}^{2}\;\pi \bar{h}$ . Since *ρ* is a function of *d*, *h*, and *μ*, which are directly measured quantities with their own uncertainties, Equation [11] applies and gives

[22] $$u_{\rho }=\bar{\rho }\sqrt{{\left(\frac{u_{\mu }}{\bar{\mu }}\right)}^{2}+{\left(2\frac{u_{d}}{d}\right)}^{2}+{\left(\frac{u_{h}}{h}\right)}^{2}}$$

By using the coverage factor *k* = 2 (which means that we multiply the result of Equation [22] by two and thus obtain a confidence level of ~95 %) and after rounding the result properly, we obtain *ϱ* = 5.58±0.08 g/cm^3^ (*R* = 1.4 %).

This exemplifies the general method of calculating the mean and uncertainty of a quantity that has not been measured directly but can be calculated from directly measured quantities.

### Estimation of uncertainties

Sometimes it occurs that repeated measurements of a quantity always give the same result. This however, does not mean that there is no uncertainty, that the measurements are prefect. The reason for always obtaining the same result is that the measurement instrument is not precise enough to record small differences. In such cases, one ought to estimate uncertainty, which then has the meaning of a maximum absolute uncertainty.

Imagine that we have the same cylinder as in the previous example. This time, we only have a scale with a precision of 0.1 g and a ruler with 1 mm spaced ticks, which is too crude to obtain anything else than *μ* = 28.9 g, *d* = 23 mm, and *h* = 12.5 mm, even if the measurements are repeated many times. Regarding the ruler, we can distinguish values with a precision of 0.5 mm, not better than that. Hence, when we say *d* = 23 mm, this actually means that *d* can be anywhere between 22.75 mm and 23.25 mm. This sets the maximum absolute uncertainty of *d* to Δ*d* = 0.25 mm, and the same applies to Δ*h* because we use the same ruler. By the same reasoning, Δ*μ* = 0.05 g. This is the usual approach to the estimation of uncertainties in cases when there is no other option.

Hence, we start with *μ* = 28.90±0.05 g, *d* = 23.00±0.25 mm, and *h* = 12.50±0.25 mm. Since *d*, *h*, and *μ* are directly measured quantities expressed through their means and maximum absolute uncertainties, we use Equation [19] to obtain

[23] $$\Delta \varrho =\bar{\rho }\left(\frac{\Delta \mu }{\bar{\mu }}+2\frac{\Delta d}{\bar{d}}+\frac{\Delta h}{\bar{h}}\right)$$

This gives $\varrho =\bar{\varrho }\pm \Delta \varrho =5.6\pm 0.2\;\text{g}/{\text{cm}}^{3}$ (*R* = 3.6 %). Note that we have not used any coverage factor, because the maximum absolute uncertainties are not related to Gaussian distribution.

It is worthwhile to mention that uncertainties are also often estimated not because a measurement instrument is not precise enough but because a detailed analysis of measurement errors is less important than keeping the presentation free of defocusing details. In any case, estimation of uncertainties always depends on the nature of an experiment, but once uncertainties have been estimated, the above procedure applies.

### Tolerances in technology

One can often see a technical report or a manual where there are expressions containing numbers and the ± sign. For instance, “the appropriate voltage for this device is 16.1±0.77 V”. Is this just another misuse of TRME, because of the inconsistent rounding of the mean and uncertainty? The answer is no, because this case is not in the domain of TRME at all. The meaning of 16.1±0.77 V is not that the applied voltage is uncertain for 0.77 V, but that the device can work properly with voltages between 15.33 V and 16.87 V. It would be equivalent to saying “you can use any voltage in the range 15.33–16.87 V, and your device will work properly”.

### Studies of statistical populations

A statistical population consists of many individual entities that differ from one another but may share a common trait that can be investigated. Investigations of statistical populations are numerous in different fields of science, such as epidemiological studies in medicine, zoological or botanical studies in biology, or geographical or geophysical studies. Related analyses of collected data may include descriptive statistics, appropriate statistical tests, comparative tests, and sometimes also TRME.

TRME is often misused in studies of statistical populations, and one can find all sorts of doubtful analyses and presentations, many containing confusing uses of *σ*, *s*, and *u*. In the Tang et al. study (7), for example, the mean height is 130.9±6.2 cm (referring to a sample of 3,194 eight-year-old boys). The meaning of this result is unclear. If 6.2. refers to *u* calculated using Equation [13], there are several problems with that. First, Equation [13] is applicable to situations where a measurement of a same sample is repeated using the same method and not to a set of measurements of different samples even if the same method is used. Second, if 6.2. is meant to represent *u*, the above result is not rounded properly, and one would also expect to see a comment on the used coverage factor. It may be, however, that 6.2 refers to *σ* or *s* (it should be *s*, because the result is related to a sample and not the entire population of eight-years-old boys in the area). In that study, the difference between *σ* and *s* is irrelevant (because n >> 1), but in some cases it might be necessary to specify it clearly. Regardless of whether it is *σ* or *s*, the notation 130.9±6.2 is wrong. Namely, 130.9 cm and 6.2 cm should be written separately, since the mean is independent of *σ* and *s*. When presenting *σ* or *s*, any reasonable number of digits can be used (there is no rounding), but the number corresponding to last digit should not be smaller than the maximum absolute error of a given measurement method.

In fact, TRME is of limited importance in studies of statistical populations, since it may account for the precision of an individual measurement but does not say much about the studied property of the whole sample/population. More information on that can be obtained in the analyses that either use a graphical presentation (8) or give the values of *x̄*, *σ* (or *s*), *α*_3_, *α*_4_, *ν*, and *η* (9).

### The mean and uncertainty of a set of independent results

Suppose that one has to analyse a set of independent results, originating from different measurements of the same quantity, and calculate the overall mean and uncertainty from these results. A good example of this situation is a calculation of the diameter *D* of a star from the measurements obtained from different observatories. The quality of these measurement may vary from observatory to observatory, since some are in a more favourable position than others or use better equipment. Each observatory provides the analyst with its result expressed as *D_i_* = *D̅ _i_*±*u_i_*.

A relatively common misuse of TRME is to calculate the mean of *D* using Equation [6] with *x*_*i*_ → *D̅ _i_*, disregard individual uncertainties *u_i_*, and then calculate *u* using Equation [13] (or even Equation [7] or Equation [9]), again with *x*_*i*_ → *D̅ _i_*. This is wrong for two reasons: 1) *D̅ _i_* is not an individual measurement of *D* in a series of the same measurements, and 2) *u_i_* carries information on the precision of each *D_i_*. This problem can be solved by using Equation [16] to calculate 〈*D*〉 and Equation [17] to calculate *u* = 〈*u_D_*〉, which have the meaning of the mean and uncertainty of *D* as calculated from the results from all of the observatories.

One may argue that the use of Equations [6] and [13] is not always completely wrong, since there is a situation when they give approximately the same result as Equations [16] and [17]. Namely, if *u_i_* ≈ *u*_0_ for every *i*, Equation [16] becomes 〈*x*〉≈ Σ_*i*_*x̄*_*i*_/*n*, where *n* is the number of independent results. However, the similarity is only numerical, and the use of Equation [6] cannot be justified conceptually. Under the same conditions, Equation [13] gives ${〈u〉}^{2}\approx u_{0}^{2}/n$ , which resembles Equation [17] if we assume *u*_0_ ≈ *s* with *x̄* → 〈*x*〉 and *x*_*i*_ → *x̄*_*i*_. Again, the similarity is only numerical and cannot be justified, especially because Equations [16] and [17] require no approximation. In addition, the statistical weight $w_{i}=1/u_{i}^{2}$ takes care that more precise results contribute more to the final result.

## CONCLUSION

The theory of random measurement errors has been used to analyse experimental data for well over a century, being mathematically complete and offering solutions for various types of analyses of measurement data. However, sometimes one can encounter partly or completely wrong usage of this theory. In particular, the mean and uncertainty of a measured quantity are often confused with the mean and standard deviation in statistics. The mean in statistics is the average value of a random variable in a statistical distribution, and in the theory of random measurement errors, it is the most probable value (the best estimate) of a measured quantity. Standard deviation in statistics is the square root of the mean squared deviation of a random variable from the mean and therefore just another statistical parameter used in the description of a statistical distribution. Uncertainty of the mean – the central quantity of the theory of random measurement errors – is conceptually different: it determines how certain we are that the mean is the best estimate of the true value of a measured quantity.
